# Applications of Biocompatible Scaffold Materials in Stem Cell-Based Cartilage Tissue Engineering

**DOI:** 10.3389/fbioe.2021.603444

**Published:** 2021-03-25

**Authors:** Xia Zhao, Daniel A. Hu, Di Wu, Fang He, Hao Wang, Linjuan Huang, Deyao Shi, Qing Liu, Na Ni, Mikhail Pakvasa, Yongtao Zhang, Kai Fu, Kevin H. Qin, Alexander J. Li, Ofir Hagag, Eric J. Wang, Maya Sabharwal, William Wagstaff, Russell R. Reid, Michael J. Lee, Jennifer Moriatis Wolf, Mostafa El Dafrawy, Kelly Hynes, Jason Strelzow, Sherwin H. Ho, Tong-Chuan He, Aravind Athiviraham

**Affiliations:** ^1^Department of Orthopaedic Surgery, The Affiliated Hospital of Qingdao University, Qingdao, China; ^2^Molecular Oncology Laboratory, Department of Orthopaedic Surgery and Rehabilitation Medicine, The University of Chicago Medical Center, Chicago, IL, United States; ^3^Department of Nephrology, The First Affiliated Hospital of Chongqing Medical University, Chongqing, China; ^4^Department of Obstetrics and Gynecology, The First Affiliated Hospital of Chongqing Medical University, Chongqing, China; ^5^Ministry of Education Key Laboratory of Diagnostic Medicine, The School of Laboratory Medicine, Chongqing Medical University, Chongqing, China; ^6^Department of Orthopaedic Surgery, Union Hospital of Tongji Medical College, Huazhong University of Science and Technology, Wuhan, China; ^7^Department of Spine Surgery, Second Xiangya Hospital, Central South University, Changsha, China; ^8^Departments of Neurosurgery, The Affiliated Zhongnan Hospital of Wuhan University, Wuhan, China; ^9^Department of Surgery, Section of Plastic Surgery, The University of Chicago Medical Center, Chicago, IL, United States

**Keywords:** articular cartilage, chondrocytes, stem cells, cartilage tissue engineering, scaffold materials, biocompatibility, osteoarthritis

## Abstract

Cartilage, especially articular cartilage, is a unique connective tissue consisting of chondrocytes and cartilage matrix that covers the surface of joints. It plays a critical role in maintaining joint durability and mobility by providing nearly frictionless articulation for mechanical load transmission between joints. Damage to the articular cartilage frequently results from sport-related injuries, systemic diseases, degeneration, trauma, or tumors. Failure to treat impaired cartilage may lead to osteoarthritis, affecting more than 25% of the adult population globally. Articular cartilage has a very low intrinsic self-repair capacity due to the limited proliferative ability of adult chondrocytes, lack of vascularization and innervation, slow matrix turnover, and low supply of progenitor cells. Furthermore, articular chondrocytes are encapsulated in low-nutrient, low-oxygen environment. While cartilage restoration techniques such as osteochondral transplantation, autologous chondrocyte implantation (ACI), and microfracture have been used to repair certain cartilage defects, the clinical outcomes are often mixed and undesirable. Cartilage tissue engineering (CTE) may hold promise to facilitate cartilage repair. Ideally, the prerequisites for successful CTE should include the use of effective chondrogenic factors, an ample supply of chondrogenic progenitors, and the employment of cell-friendly, biocompatible scaffold materials. Significant progress has been made on the above three fronts in past decade, which has been further facilitated by the advent of 3D bio-printing. In this review, we briefly discuss potential sources of chondrogenic progenitors. We then primarily focus on currently available chondrocyte-friendly scaffold materials, along with 3D bioprinting techniques, for their potential roles in effective CTE. It is hoped that this review will serve as a primer to bring cartilage biologists, synthetic chemists, biomechanical engineers, and 3D-bioprinting technologists together to expedite CTE process for eventual clinical applications.

## Introduction

**FIGURE 1:**
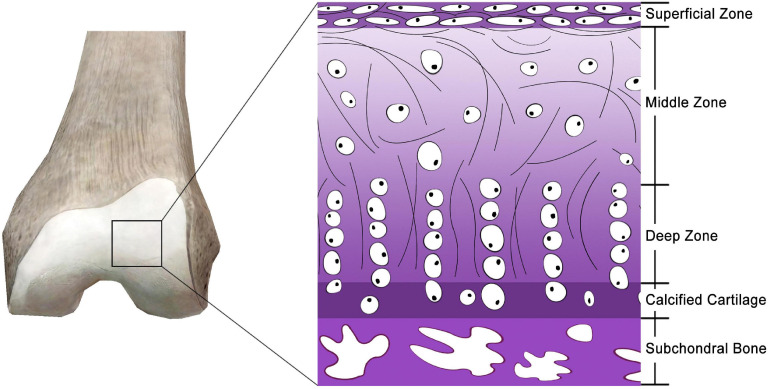
Schematic depiction of articular cartilage and chondrocytes of the joint surface.

Articular cartilage, also known as hyaline cartilage, is a unique and durable connective tissue that plays a critical role in physiological mobility by providing nearly frictionless articulation for mechanical load transmission between joints (Figure 1) (Ge et al., 2012). Owing to the scarcity and poor proliferative activity of adult chondrocytes, a lack of vascularization and innervation, a slow matrix turnover, and a low supply of progenitor cells (Hunziker, 2002; Makris et al., 2015), articular cartilage has a very low intrinsic self-regeneration capacity after injury (Moura et al., 2020). Furthermore, the chondrocytes of articular cartilage are entrapped in a low-nutrient and low-oxygen environment (Yamagata et al., 2018). Damage to the articular cartilage frequently results from sport-related injuries, diseases, degeneration, trauma, and tumors. Failure to treat impaired cartilage may lead to osteoarthritis (Muzzarelli et al., 2012), the most common joint disease responsible for pain and disability affecting over a quarter of the adult population (Chen et al., 2017). Cartilage damage usually recovers through scar tissue formation that is primarily composed of fibrocartilage (Ahmed and Hincke, 2010). Hence, it is essential to explore new techniques for articular cartilage regeneration to effectively restore the function of joints.

Ideal cartilage repair aims to restore key properties of the original hyaline cartilage in terms of histological structure and biomechanical functions, which can be only achieved by replacing with healthy cartilage tissue (Alkaya et al., 2020). However, current treatments, including microfracture, autologous and allogeneic osteochondral transplantation, and autologous chondrocyte implantation (ACI), frequently result in the formation of fibrocartilage tissue rather than the ideal native-like hyaline cartilage, leading to serious adverse effects including pain, donor site morbidity, inconsistent long-term effects, and infection (Levy et al., 2013; Solheim et al., 2013; Valderrabano et al., 2017; Moura et al., 2020). ACI is a technique wherein chondrocytes harvested and cultured from healthy cartilage are embedded onto defected cartilage covered with periosteum (Alkaya et al., 2020). The resultant cartilage-like tissue can be integrated with surrounding normal cartilage and function mechanically following regeneration. Currently, ACI is a prevalent clinical method for the treatment of articular cartilage defects, with positive outcomes in patient satisfaction and standard knee scores at short- and mid-term follow-ups (Bentley et al., 2003; Mandelbaum et al., 2007).

While ACI shows promising results in producing hyaline-like tissue (Minas et al., 2016; Sheu et al., 2017; Krill et al., 2018), several drawbacks include donor site morbidity, a shortage in the supply of chondrocytes, chondrocyte dedifferentiation in monolayer culture, and periosteal hypertrophy (Steinwachs and Kreuz, 2007). The biggest challenge of ACI is that chondrocytes are highly prone to transforming into fibroblasts and age considerably faster under *in vitro* conditions (Gosset et al., 2008). Moreover, implantation of chondrocytes without proper scaffolds often leads to a high likelihood of graft failure, delamination, and tissue hypertrophy (Wood et al., 2006). The low quantity of available cartilage cells, further compounded by their low mitotic activity level, results in difficulty obtaining sufficiently large amounts from cellular cultures (Zylinska et al., 2018; Alkaya et al., 2020). Collectively, while cartilage restoration techniques such as osteochondral transplantation, ACI, and microfracture have been used to repair certain cartilage defects, the overall clinical outcomes are often mixed and undesirable. Better and more efficient cartilage injury repair approaches have to be devised.

Cartilage tissue engineering (CTE) may hold promise to facilitate cartilage repair. Ideally, a successful CTE requires at least three critical parameters: the use of effective chondrogenic factors, an ample supply of chondrogenic progenitors, and the employment of cell-friendly, biocompatible scaffold materials (Green et al., 2015; Mostafa et al., 2019). For the past 10 years, significant progress amounts of basic and translational research have identified biofactors that can promote and/or facilitate chondrocyte differentiation and cartilage maturation, while various sources of mesenchymal stem cells (MSCs) and/or induced pluripotent stem cells (iPSCs) have been characterized as potential chondrocyte progenitor cells (Castro-Vinuelas et al., 2018; Rim et al., 2018). The advent of three-dimensional (3D) bio-printing should further facilitate the progress of CTE (Bishop et al., 2017).

In this review, we briefly discuss potential sources of chondrogenic progenitors, mostly MSCs. We then emphasize on currently available chondrocyte-friendly scaffold materials, including natural and synthetic polymers and extracellular matrix, along with 3D bioprinting techniques, for their potential roles in effective CTE. Whenever possible, we highlight promising results from *in vitro* and/or *in vivo* studies involved in the uses of biofactor-stimulated progenitor cells delivered with biocompatible scaffold materials in this review. It is hoped that the review can serve as a primer to bring cartilage biologists, synthetic chemists, biomechanical engineers, and 3D-bioprinting technologists together to expedite the CTE process for eventual clinical applications.

## Mesenchymal Stem Cells (MSCs) as an Important Chondrogenic Progenitor Source for Cartilage Tissue Engineering (TE)

Ideal progenitor cell sources to replace chondrocytes should have the following features: a pool of undifferentiated cells featuring high regenerative potential, unlimited division capacity, self-renewal capability, easy accessibility, and hypo-immunogenicity (Li Y.Y. et al., 2014). MSCs are multipotent progenitor cells that can self-renew and differentiate into several lineages including bone, cartilage, fat, and muscle (Green et al., 2015; Pittenger et al., 2019; Gomez-Salazar et al., 2020). While the most common used adult source tissues for human MSCs are bone marrow and adipose tissue, MSCs have been identified in numerous connective soft tissues (Pittenger et al., 2019; Gomez-Salazar et al., 2020). As one of the most popular progenitor sources, MSCs have been used in nearly 1,000 clinical trials for diverse indications, ranging from musculo-skeletal defects, disorders of the immune system including auto-immune diseases, to myocardial infarcts (Rastegar et al., 2010; Teven et al., 2011; Beederman et al., 2013; Coalson et al., 2019; Pittenger et al., 2019; Gomez-Salazar et al., 2020; Pakvasa et al., 2021). Nonetheless, MSCs remain a biological enigma, since retrospective derivation in culture has concealed the true native identity of these cells, so their roles in tissue regeneration remain to be fully understood (Gomez-Salazar et al., 2020).

During *in vivo* cartilage repair experiments, MSCs showed improved cell arrangement, subchondral bone regeneration, and integration compared to mature chondrocytes, suggesting that MSCs are a suitable alternative for cartilage repair (Li Y.Y. et al., 2014). Although MSCs can be injected intravenously (IV), intra-articularly (IA), or intraperitoneally (IP), the cells ultimately diffuse into the peripheral blood and occupy the non-affected area (Gonzalez et al., 2009; Ra et al., 2011; Liang et al., 2012; Zhang et al., 2014). Efforts have been attempted to transplant MSCs formed in 3D structures, such as seeding in scaffolds, cell aggregates, and sheets (Tasso et al., 2009). Thus, tissue engineering (TE) provides a promising approach for cartilage restoration using MSCs (Chung and Burdick, 2008). The ultimate goal of TE is to develop biological substitutes that can be implanted into the body, supporting tissue remodeling in the frame of a 3D scaffold (Kim W.K. et al., 2019).

The delivery and implantation of MSCs into cartilage defects can be accomplished by seeding MSCs onto different types of scaffolds, which are then introduced into cartilage lesions. An ideal scaffold should contain implanted MSCs and bioactive molecules for chondrocyte differentiation and maturation (Henderson et al., 2004). Growth factors are prime bioactive molecules capable of inducing chondrogenic differentiation of MSCs (Peterson et al., 2000). A major challenge in TE in 3D microenvironments is the need for biomaterial scaffold to promote cell attachment, spreading, migration, proliferation, and differentiation for effective tissue regeneration (Im, 2020; Mohammadinejad et al., 2020). Such biomaterials should have the same mechanical properties as native cartilage, be able to properly integrate with adjacent cartilage, be porous with interconnected pores, have the ability to establish and maintain the desired shape of the regenerated cartilage, and yet adequately biodegrade (Freedman and Mooney, 2019; Alkaya et al., 2020). In the following sections, we summarize the features of commonly used scaffold materials, and preparation techniques for biological scaffolds utilized in either experimental or clinical settings to develop TE for cartilage repair.

## Natural Polymers as Scaffolds for Cartilage Tissue Engineering

Natural polymers, such as chitosan, collagen, alginate, silk fibroin, hyaluronan, and gelatin, have been used extensively in TE for cartilage regeneration (Table 1). Due to their superior biocompatibility, excellent biodegradability, minute negative immunological influence, and favorable cellular interaction (Alkaya et al., 2020), many natural materials have been employed to provide a satisfactory bioactive environment and mechanical support to foster the growth of new chondral tissue at defect sites (Bharadwaz and Jayasuriya, 2020). Natural polymers show structural compatibility akin to the biological molecules found in organisms when implanted *in vivo*, thus reducing the risk of an immune response (Mohammadinejad et al., 2020). Consequently, certain polysaccharides are either non-immunogenic or possess low immunogenicity when compared with synthetic polymers. Moreover, ligands of natural biomaterials can facilitate cell adhesion and promote the activation of various chondrogenic activation pathways (Alkaya et al., 2020).

**TABLE 1:** Characteristics of the outlined natural polymers for CTE.

Biomaterials	Characteristics	Advantages	Disadvantages	References
Chitosan	Originating from chitin; Linear natural carbohydrate biopolymer; Free amine groups in its backbone chain; Slower degradation rate	Biodegradability; Biocompatibility; Non-antigenicity; Adsorption capabilities; Antimicrobial activity; Promoting chondrogenesis	Low solubility; Low mechanical strength	Keller et al. (2017), Giuliani (2019), Sultankulov et al. (2019)
Collagen	Important part of natural cartilage organic materials; One of the most abundant proteins in humans and a major component of extracellular matrix	Biocompatibility; Low immunogenicity; Biodegradability; Promoting chondrogenesis; Facilitation of cell ingrowth and remodeling; Easy processing	Low solubility; Low mechanical strength; Rapid biodegradation rate	Lee et al. (2001), Kuroda et al. (2007), Turk et al. (2018), Li L. et al. (2019), Marques et al. (2019)
Silk	Extracted from Bombyx mori cocoon; A biocompatible material found as the core of a structural protein fiber;	Excellent mechanical properties; Biocompatibility Controlled biodegradability; Lower infection risk; Easy processing;	Delayed hypersensitivity; Initiator of immune reactions;	Zhang et al. (2010), Wang et al. (2011), Ma et al. (2018), Bharadwaz and Jayasuriya (2020)
Alginate	Produced from the cell wall of brown algae; Polysaccharide with negative charge; A cell-friendly gelation	Low immunogenicity; Biocompatibility; High abundance resources; Low prices; Regulation of the inflammatory chemokines; Good chondrogenic potential	Low biodegradability; Poor adhesion	Cho et al. (2009), Arlov et al. (2014), Park and Lee (2014), Filardo et al. (2018), Li L. et al. (2019)
Hyaluronic acid	A disaccharide unit; Abundant in the human body, present in the ECM of the skin, cartilage, and lenses	Biocompatibility; High hydrophilicity; Nontoxicity; Elasticity; Anti-inflammatory	Low mechanical properties; Rapid enzymatic degradation	Collins and Birkinshaw (2013), Gupta et al. (2019), Li L. et al. (2019), Zheng et al. (2019)
Gelatin	Obtained from native collagen via hydrolysis; An ideal carrier of proteins, growth factors, and so on	Biocompatibility; Biodegradability; High water-solubility; Cell adhesion	Poor mechanism properties	Larsen et al. (2006), Li F. et al. (2017), Echave et al. (2019)
Platelet-rich fibrin	Derived from platelet-rich plasma; Second-generation platelet concentrate containing abundant growth factors	Greater quantities of growth factors; Outstanding handling and storage traits; Low prices; Easy preparation	Poor mechanism properties	Miron et al. (2017), Wong et al. (2017), Wu et al. (2017), Barbon et al. (2019)
Cellulose	Durable, fibrous, and water-insoluble substance from plant cell walls	Biodegradability; Biocompatibility; Outstanding mechanical properties; Non-toxic Low prices; Natural abundance	Poor mechanism properties	O’Sullivan (1997), Hubbe et al. (2017), Isobe et al. (2018), Tayeb et al. (2018), Dutta et al. (2019)

### Chitosan

Chitosan (CS) has emerged as a strong candidate scaffold for TE applications. Originating from chitin, chitosan is a unique natural polysaccharide with superb properties: high biodegradability, biocompatibility, non-antigenicity, adsorption capabilities, and antimicrobial activity (Giuliani, 2019). Studies have also found no complications, such as inflammation or allergic reactions, following implantation of CS-based scaffolds (Keller et al., 2017). Due to the existence of free amine groups in its backbone chain, CS can be further modified chemically to introduce useful properties for biomaterial development (Sultankulov et al., 2019). Its hydrophilic structure promotes cell adhesion, proliferation, and differentiation, while its polycationic structure at mild acidity allows for gene delivery through the immobilization of negatively charged DNA, proteins, and enzymes (Sultankulov et al., 2019).

Chitosan is a linear natural carbohydrate biopolymer with configurational similarity to glycosaminoglycans of the ECM for cell–cell adhesion (Rodriguez-Vazquez et al., 2015). CS can present a similar microenvironment, allowing for chondrocyte proliferation and thus inducing chondrogenesis and ECM synthesis (Liu et al., 2017). Its chemical name is (1,4)-2-amino-2-deoxy-beta-D-glucan, a copolymer of randomly located (1→4)-2-amino-2-deoxy-d-glucan (d-glucosamine) and (1→4)-2-acetamido-2-deoxy-d-glucan (*N*-acetyl d-glucosamine) units (Sultankulov et al., 2019). CS degrades at a slower rate compared to other natural polymers, such as fibrin, gelatin, and collagen (Sultankulov et al., 2019). The physical properties of CS are dependent on its the molecular weight, degree of polymerization, and purity of the product (Tharanathan and Kittur, 2003). Shortcomings of pure CS, especially its mechanical strength, can be rectified by the formulation of CS-based nanocomposite scaffolds, mainly with tricalcium phosphate, collagen, hydroxyapatite, and synthetic polymers (Bharadwaz and Jayasuriya, 2020).

Chondrocytes cultured in CS-alginate beads were shown to express lower levels of inflammatory cytokines (IL-6 and IL-8) and higher levels of cartilage matrix component genes (hyaluronan and aggrecan) *in vitro* when compared to alginate beads alone (Bhattacharjee et al., 2016). The addition of hyaluronic acid-CS nanoparticles (NPs) to a pellet co-culture of human infrapatellar fat pad (IPFP)-derived MSCs with osteoarthritic chondrocytes led to greater levels of chondrogenic differentiation (Datta et al., 2001). Human IPFP-MSCs seeded on 3D-printed CS scaffolds in chondrogenic media containing TGF-β3 and BMP-6 attached, proliferated, and differentiated into chondrocyte-like cells in the formation of cartilaginous tissue *in vitro* (Patra et al., 2012). Chitosan was shown to induce human bone marrow MSCs to differentiate into chondroid spheres by activating mTOR/S6K (Li S. et al., 2019). A chitosan–hyaluronic acid-based biometric matrix was shown to provide an appropriate environment, allowing adipose-derived stem cell (ASC) differentiation into cartilage matrix producing chondrocytes (Huang et al., 2019).

Chitosan (CH), poly (L-lactide) (PLLA), and pectin (PC) compositions have been adapted using the freezing drying method to create polyelectrolyte complex-based porous scaffolds, then crosslinked using 1-ethyl-3-(3-dimethylaminopropyl) carbodiimide (EDC) and a *N*-hydroxysuccinimide (NHS) solution containing chondroitin sulfate (CS) to mimic the composition and architecture of the cartilage ECM (Mallick et al., 2018). This type of scaffolds exhibited a satisfactory swelling profile and moderate biodegradation, as well as being hemocompatible with sufficient mechanical strength for applications in cartilage tissue regeneration. In order to investigate whether the chitosan gel can adhere to cartilage and bone in various animal bone defects, the a space-filling and cyto-compatible chitosan gel solution was designed and shown to adhere to cartilage and bone *in situ*, a property that indicated high potential for its use as an arthroscopically injectable vehicle for cell-assisted cartilage repair (Hoemann et al., 2005).

### Collagens

Collagens (COL) are one of the most abundant proteins in humans and a major component of the ECM and have superb biocompatibility, low immunogenicity, interactivity with growth factors and cell adhesion molecules, biodegradability, and facilitation of cell ingrowth and remodeling (Lee et al., 2001). The ECM of articular cartilage is composed of approximately 90% type II collagen (Spratt and Haycock, 1988). Collagens are comprised of polypeptide chains composed of various amino acids, typically in the tripeptide sequence glycine X-Y (X and Y are frequently proline and hydroxyproline) (Marques et al., 2019), forming a triple helix structure promoting collagen’s structural stability and excellent mechanical properties (Silva et al., 2014).

Collagens are found in abundant quantities from fish waste, such as skins, scales, and bones (Senaratne et al., 2006; Ge et al., 2012). Aquatic sources, such as cuttlefish (Nagai et al., 2001), jellyfish (Song et al., 2006), the skin and muscles of oceanic animals, and fish waste (Mahboob, 2015) are regarded as superior to bovine sources (Silva et al., 2014), as there is less concern over the potential transmission of spongiform encephalopathy (Senaratne et al., 2006). Nonetheless, the rapid biodegradation rate of pure collagen scaffolds, their low mechanical strength, and their tendency to cause frequent swelling incentivizes the use of collagen-based composite biomaterials for CTE (Turk et al., 2018).

Collagen-based materials support chondrocyte differentiation and are frequently used in the repair of articular cartilage (Li Y.Y. et al., 2014). When rabbit MSCs and collagen were encapsulated as microspheres, and implanted into the osteochondral defects in an animal model the scaffold promoted spontaneous differentiation of endogenous MSCs into chondrocytes (Yamagata et al., 2018). The implantation of collagen gel and MSCs into an athlete suffering from knee pain resulted in hyaline-like tissue formation and functional recovery of the articular cartilage (Kuroda et al., 2007). The mixture of rabbit chondrocytes with rabbit and rat collagen scaffolds to form neo-RBT (neo-rabbit cartilage) and neo-RAT (neo-rat cartilage) constructs featured cartilage-like repair tissue covering the 5-mm circular, 4-mm deep defects created in the rabbit condyles (Wang et al., 2018).

Collagens exhibit several advantageous characteristics for drug delivery, including high biodegradability and biocompatibility, low toxicity, high efficiency, and a long period of effectiveness (Li L. et al., 2019). However, the interaction level between enzymes and other bioactive substances is weaker in collagen than in hydrophobic polymers (Li L. et al., 2019). Chondrocytes embedded in the hydrogel with type I and II collagens maintained their natural morphology and secreted cartilage-specific ECM, which could be altered by changing the amount of type I collagen (Yuan et al., 2016). Hydrogel formed from HA and type II collagen are also able to form *in situ* scaffolds. Chondrocytes and TGFβ1, encapsulated in the scaffold, maintained chondrocyte viability and stimulated glycosaminoglycan production, gene expression, and cell proliferation and morphology (Kontturi et al., 2014).

Collagen can also be used as a bioink component of 3-D bio-printing for CTE applications (Kim et al., 2016; Lee et al., 2018; Yang X. et al., 2018). When three different combinations using 3D bioprinting: alginate (SA), alginate/agarose (SA/AG) and alginate/collagen (SA/COL), were tested, the SA/AG bioinks achieved superior tensile strength and compressive modulus, while the SA/COL bioinks featured the best cell viability of the three, as indicated by higher levels of several specific cartilage gene markers (Yang X. et al., 2018). The collagen-based bioinks also led to significantly higher expression levels of specific osteogenic gene markers for human adipose stem cell (hASC) differentiation (Kim et al., 2016). Similarly, Lee et al. (2018) investigated the production of cell-laden collagen structures with a bioink container, and did not find any problems associated with collagen printing when using the most optimal parameters described in the previously mentioned work.

### Silk Fibroin

Silk fibroin (SF) is one of the oldest natural polymers and considered an enticing polymer for various biomedical applications, with an evolutionary history spanning over 380 million years (Ma et al., 2018). Extracted from *Bombyx mori cocoon*, a mulberry source, SF is a biocompatible material found at the core of a structural protein fiber that is coated with sericin, and has been used in several tissue engineering applications (Bharadwaz and Jayasuriya, 2020). SF extracted from non-mulberry sources, such as the *tasar silkworm* (*Antheraea mylitta*), has improved mechanical properties compared to SF isolated from mulberry sources (Kundu et al., 2012). SF-based biomaterials have several advantages over other natural polymers derived from tissues of allogeneic or xenogeneic origins. SF-based biomaterials have a lower infection risk and lower costs due to less complex processing procedures (Ma et al., 2018). Silk fiber purification is typically performed with a simple alkali or enzyme-based degumming protocol, which results in fibrin without sericin (Ma et al., 2018). SF also benefits from the large-scale processing infrastructure already established by traditional silk textile industries, further lowering costs (Kundu et al., 2013).

However, certain foreign body responses can be triggered by SF, reminiscent of non-autologous biomaterials of non-mammalian origin (Giesa et al., 2011). It has been suggested the delayed hypersensitivity of silk sutures may relate to the presence of sericin (Du et al., 2011). Nonetheless, further studies must be done to identify the specific source(s) of any immunogenic remnants in silk (Zhang et al., 2010). The biocompatibility of SF-based materials has been well tested when applied in musculoskeletal tissue engineering (MTE) (Meinel and Kaplan, 2012). It was reported that SF 3-D scaffolds activated very mild immune responses after subcutaneous implantation in rats over a period of 1 year; and all genes associated with immune response, including TNF-α, IFN-δ, IL-4, IL-6, and IL-13, were held at undetectable expression levels for most types of silk sponges (Wang et al., 2008).

A proper balance of mechanical properties such as breaking strength, modulus, and elongation can make silk a tough, ductile, and attractive material (Vollrath and Knight, 2001). Silk has a strength-to-density ratio up to ten times higher than steel (Gellynck et al., 2008). Such outstanding mechanical qualities provide many potential applications of SF-based materials: its high tensile strength makes it applicable for sutures, while its flexibility is suitable for creating loadbearing scaffolds (Altman et al., 2002). In addition to its good extensibility range, elasticity, strength, and strain hardening, silk’s mechanical behavior can also be modified and tuned by altering the protein concentrations and the size and density of pores (Nazarov et al., 2004; Kim et al., 2005).

When implanted, SF products have variable degradation rates determined by the secondary structure of silk formed during regeneration (Wang et al., 2011). Wang et al. (2008) found that water based porous SF scaffolds implanted in rats disintegrated and completely disappeared after 1 year. They also reported silk was bio-resorbable in addition to being biodegradable, suggesting that the host immune system causes degradation of silk and silk material-based scaffolds (Wang et al., 2008). Sengupta et al. (2010) detailed that osteoclasts and osteoblasts were able to invade SF films by expressing metalloproteinases (MMPs). Unlike synthetic biomaterials with faster degradation and less desirable mechanical properties, SF systems are better suited for TE because of their ability to retain strength over extended periods of time *in vivo*, a trait that is essential given the need for slow degradation and load bearing capacity in TE (Ma et al., 2018). Nonetheless, a truly comprehensive understanding of degradation and the clearing mechanisms of silk prescribes additional investigation, which may benefit the continued development of SF as biomaterial scaffolds (Ma et al., 2018).

### Alginate

Alginate is a natural polysaccharide composed of 1,4-linked D-mannuronic acid (M-block) and L-guluronic acid (G-block) residues (Li L. et al., 2019). It can be harvested from the cell wall of brown algae, and is widely used in TE due to its low immunogenicity, high biocompatibility and availability, and cell-friendly gelation (Rehm and Valla, 1997). Due to these traits, along with its efficient complexation with divalent cations and highly hydrated viscoelastic properties, alginate has been widely implemented to enfold various types of cells into hydrogels (Cho et al., 2009). It has been approved by the US Food and Drug Administration (FDA) for human use as a food additive and wound dressing material. Alginate has a low cost, no cellular toxicity, and easy fabrication of 3D porous scaffolds or cell immobilized beads. However, alginate-only scaffolds showed poor adhesion to anchor-dependent cells (Zehnder et al., 2015). Furthermore, it retains long-term *in vivo* stability since mammals do not express alginate lyases or other known enzymes with homologous functions, and thus cannot degrade alginate (Lee and Mooney, 2012).

The sulfation of alginate increases its negative charge, promoting electrostatic interactions typical of sulfated GAGs (Arlov et al., 2014). From a biochemical perspective, sulfated alginate is a heparin/heparan sulfate analog, which interacts with heparin-binding proteins to inhibit both inflammatory pathways and complement activation (Kerschenmeyer et al., 2017). Moreover, sulfated alginate hydrogels were used as a mitogenic signaling scaffold to induce chondrocyte expansion, while preserving the native cartilage phenotype (Kerschenmeyer et al., 2017).

Alginate is a propitious biomaterial for scaffold-based approaches, possessing good chondrogenic potential and superb biocompatibility (Filardo et al., 2018). Alginate is also dimensionally stable and supports chondrogenic differentiation due to the absence of adhesive domains that may inhibit chondrogenesis (Ma et al., 2012). Alginate gel can be chondroinductive following the embedding of hMSCs (Diduch et al., 2000). Alginate can undergo gentle gelation with multivalent cations like Ca^2+^, producing hydrogels that showed excellent biocompatibility (Ko et al., 2010). When combined, alginate microspheres and HA hydrogel serve as a composite carrier of MSCs as well as transforming growth factor (TGF) and retains its bioactivity in the scaffold, promoting chondrogenesis of MSCs (Bian et al., 2011). Although it possesses a lower elastic modulus than normal alginate-based hydrogel, the oxidized alginate hydrogel has a higher capability for cartilage repair (Bouhadir et al., 2001). Blending the arginine-glycine-aspartic acid (RGD)-modified oxidized alginate, hyaluronate and chondrocytes formed an injectable hydrogel, which expressed the chondrogenesis-related protein and chondrogenic marker gene at 6 weeks after injection (Park and Lee, 2014).

### Hyaluronan Acid/Hyaluronan

Hyaluronan, also known as hyaluronic acid (HA), is a disaccharide unit composed of *N*-acetylglucosamine and D-glucuronic acid (Li L. et al., 2019). It is abundant in the human body, present in the ECM of the skin, cartilage, and lenses. HA does not contain sulfur like other mucopolysaccharides, and the molecular weight varies widely in different tissues. As a major component of the ECM, HA supports cell migration, proliferation, and morphogenesis (Collins and Birkinshaw, 2013). HA can also provide cells with a 3D microenvironment closely resembling natural conditions (Li L. et al., 2019), and plays a significant role in wound healing and cell signaling (Toole, 2004). Chondrocytes can firmly attach to such hyaluronan-based matrices (Mohammadinejad et al., 2020). HA also binds to specific receptors expressed in many cells, triggering several intracellular signal events (Cao et al., 2005).

Hyaluronic acid has been used since the 1970s in humans to treat joint pain and other health conditions (Gupta et al., 2019), as well as in various applications such as tissue engineering, regenerative medicine, and clinical practice (Li L. et al., 2019). A chitosan–hyaluronic acid-based biomimetic matrix when used in conjunction with ASC was shown to form articular hyaline cartilage (Huang et al., 2019). It was reported that platelet-rich plasma (PRP), rich in various cytokines, proteins, and growth factors, was combined with HA hydrogel to repair critical-size focal cartilage defects in porcine condyles (Yan W. et al., 2020). Exogenous HA prevents the degradation of cartilage while promoting its regeneration and enhancing chondrocyte HA synthesis, reduces proinflammatory mediator production, and suppresses matrix metalloproteinases involved in OA pathogenesis (Li L. et al., 2019). HA gels combined with proteoglycan may be suitable for use as an injectable therapeutic agent, delaying or inhibiting OA onset following knee injuries (Srinivasan et al., 2012). HA possesses several advantages such as high hydrophilicity, nontoxicity, and biocompatibility (Zheng et al., 2019). Under typical shear rates across two sliding surfaces of articular cartilage, HA viscosity decreased dramatically and became comparable to water due to the shear-thinning effect, ruling out its application for joint lubrication (Jahn et al., 2016). HA degrades *in vivo* due to it being a natural polymer and also displays variable lubrication and anti-inflammatory capability based on molecular weight (Zheng et al., 2019).

### Gelatin

Gelatin is a fibrous protein composed of a unique sequence of amino acids obtained from native collagen via hydrolysis. Gelatin exhibits good biodegradability and biocompatibility (Aldana and Abraham, 2017), and possesses the ability to create poly-ionic complexes with charged therapeutic compounds such as polysaccharides, growth factors, proteins, and nucleotides (Larsen et al., 2006), which make gelatin an idea delivery vehicle for a variety of biomolecules (Echave et al., 2019). When MSCs were implanted into rabbit osteochondral defects, gelatin and MSCs were found to be highly biocompatible, without evidence of immune response or lymphocytic infiltration at the site (Yamagata et al., 2018). Gelatin can be used in the construction of scaffolds to improve cell adhesion, infiltration, spread, and proliferation (Sajkiewicz and Kolbuk, 2014).

Gelatin methacryloyl (GelMA) is produced through the reaction of gelatin with methacrylic anhydride (MA) (Van den Bulcke et al., 2000). GelMA hydrogels are notably similar to the ECM, and the mechanical, swelling, and lubricating properties of GelMA hydrogels are reminiscent of natural cartilage (Spiller et al., 2011). Microporous GelMA hydrogels displayed higher rates of proliferation, while GelMA hydrogels without a microporous structure possessed significant advantages in the cartilaginous phenotype (Li X. et al., 2017). A study over the impact of spatial chondrocyte distribution on cartilage defect repair indicated that spatial chondrocyte distribution indeed served an important role in the repair process (Mouser et al., 2018).

Gelatin-based 3D microgels can be utilized to stimulate cell proliferation and bolster the differentiation of encapsulated cells such as stem cells (Li F. et al., 2017). These microgels are capable of shielding the cells from shear-force associated mortality during injection and provide them with a milieu that enhances cell retention within the targeted site (Nichol et al., 2010). Injectable covalently cross-linked gelatin hydrogels have been created recently with the assistance of pendant tetrazine or norbornene click chemistry pairs in modified polymers (Koshy et al., 2016). These gelatin polymers rapidly crosslink together and begin to degrade after injection *in vivo*, while they facilitate cell viability and transform encapsulated cells into 3D elongated morphologies (Echave et al., 2019). A thermoresponsive gelatin, poly(*N*-isopropylacrylamide)-grafted gelatin (PNIPAAm–gelatin) was found suitable as *in situ* formable scaffold for cartilage repair (Ibusuki et al., 2003).

Natural and synthetic polymeric electrospun scaffolds have recently gained attention due to their ability to mimic the ECM. One pertinent electrospun fibrous membrane is a hybrid of gelatin and polycaprolactone (GT/PCL), a versatile biomimetic substrate for soft tissue engineering including cartilage (Xue et al., 2013; Zheng et al., 2014). Xue et al. (2013) and Zheng et al. (2014) developed a sandwich model, in which cells were seeded on acellular cartilage sheets layer-by-layer over a titanium alloy mold to generate ear-shaped cartilage. The engineered 3D cartilage using the sandwich model and GT/PCL 70:30 electrospun fibrous membranes proved to be effective (Xue et al., 2013; Zheng et al., 2014). Scaffolds produced using this method also possess superb *in situ* space-filling qualities in both air and aqueous solutions, without the use of protective barriers. In a separate study, Lin et al. (2019) supplemented mGL scaffolds with bioactive polymers like HA to further optimize them. The ideal ratio was 9:1 of mGL:mHA, generating the best cartilage with high levels of chondrogenesis. The mGL/mHA (9:1) scaffolds also induced bone and cartilage generation after 12 weeks following implantation into rabbit osteochondral defects, highlighting potential future clinical applications (Lin et al., 2019).

### Platelet-Rich Fibrin

Autologous platelet concentrates allow high local concentrations of growth factors while remaining low in cost and complexity. Derived from PRP, platelet-rich fibrin (PRF) is a second-generation platelet concentrate containing abundant growth factors such as fibroblast growth factors (FGFs), platelet-derived growth factors (PDGFs), epidermal growth factor (EGF), insulin-like growth factors (IGFs), TGFs, and vascular endothelial growth factors (VEGFs) Wu et al. (2017). Kobayashi et al. (2016) compared the release of growth factors for PRF, PRP, and advanced platelet-rich fibrin (A-PRF), finding that PRP exhibited significantly higher release levels at earlier time points. PRF exhibited a steady release over a 10-day period. A-PRF released significantly greater quantities of growth factors than traditional PRF (Miron et al., 2017). Thus, PRP is optimal for fast delivery of growth factors while PRF and A-PRF are best used when long-term release is desired (Grecu et al., 2019). PRF is composed of leukocytes, cytokines, platelets, and adhesive proteins such as fibronectin, fibrinogen, vitronectin, and thrombospondin-1 (Miron and Zhang, 2018). This blood-derived membrane is also enriched with leukocytes that play a key role in antibacterial immune responses, contributing to wound healing (Dohan et al., 2006; Fioravanti et al., 2015).

Platelet-rich fibrin has drawn attention due to its potential benefits for tissue injury and would healing (Barbon et al., 2019). The affordability, low risk to patients, and ease of preparation all contribute to PRF’s status as an ideal scaffold for tissue healing (Maia et al., 2018). Fibrin polymerization results in a 3D cross-linked fibrin matrix (Fioravanti et al., 2015) that can serve as a binding site for growth factors and platelets (Caloprisco et al., 2010; Caloprisco and Borean, 2011). Thus, PRF enhances tissue regeneration by raising growth factor concentration and mimicking the natural process of tissue repair over time (Di Liddo et al., 2018; Miron and Zhang, 2018).

Platelet-rich fibrin was shown to augment proliferation, chemotaxis, and viability of chondrocytes, and induced chondrogenic differentiation in cultured chondrocytes as the expression of markers such as aggrecan and type II collagen was detected (Wong et al., 2017). PRF improved formation and deposition of cartilaginous matrix by cultured chondrocytes (Wong et al., 2017). Spreafico et al. (2009) found that human platelet releasates bolstered ECM synthesis and deposition while maintaining the normal phenotype of chondrocytes. PRF releasate (PRFr) was recently derived from human bloods (Su et al., 2009), and the concentrations of lipids, proteins, and growth factors were higher in PRFr compared to supernatant serum (Burnouf et al., 2012). PRFr was found to upregulate the expressions of aggrecan and type II collagen, heightening the production of proteoclycan and glycosaminoglycan in human OA chondrocytes (Wu et al., 2017). PRF generated a favorable environment for stem cell differentiation and proliferation due to the release of endogenous growth factors (Kazemi et al., 2017).

Platelet-rich fibrin has emerged as a promising biological tool for cartilage regeneration as it carries supraphysiological levels of cytokines and growth factors to injury sites (Barbon et al., 2019). The *in situ* administration of PDGFs was shown to stimulate *in vitro* chondrocyte differentiation and proliferation (Brandl et al., 2010), along with promoting *in vivo* cartilage healing (Fortier et al., 2011). Gaissmaier et al. (2008) reported higher human chondrocyte proliferation rates resulting from the addition of 1% and 10% human platelet supernatant in culture, and Akeda et al. (2006) reached a similar conclusion regarding the stimulating effect of 10% PRP-enriched medium on porcine chondrocyte proliferation (Akeda et al., 2006). Since PRF also contains a multitude of platelet-derived cytokines and growth factors, PRF may be capable of promoting articular cartilage regeneration, providing suitable mechanical properties (Barbon et al., 2019).

Platelet-rich fibrin has recently emerged as a promising non-surgical means to treat cartilage injuries. Chien et al. (2012) were among the first to demonstrate PRF inclusion in biodegradable fibrin scaffolds as a regeneration matrix to support chondrocyte proliferation and redifferentiation. A culture-free, single-stage approach has been developed, combining PRF with autologous cartilage grafts and negating the need for complex procedures involving *in vitro* chondrocyte expansion (Wu et al., 2017). In a clinical study, a polymer-based implant was combined with PRF glue and used to treat patients (McDermott, 2019). The procedure was safe and suitable for patients suffering from full-thickness chondral lesions on the patella resulting from microfractures (McDermott, 2019). Thus, PRF prepared from autologous origins to reduce pathogen transmission and immune rejection risks may open the door for its use in regenerative medicine (Barbon et al., 2019).

### Cellulose

Cellulose is a durable, fibrous, and water-insoluble substance from plant cell walls (Dutta et al., 2019), although it can be also produced by some animals (e.g., tunicates), fungi, and bacteria (O’Sullivan, 1997; Eichhorn et al., 2005). Some bacterial genera, such as *Pseudomonas, Agrobacterium, Gluconacetobacter, Sarcina*, and *Rhizobium* can synthesize bacterial cellulose (BC) from glucose and other carbon sources (Dutta et al., 2019). Bacterial cellulose has been tested as naturally occurring ‘nanomaterial’ scaffolds (Dutta et al., 2019). The micro-crystalline structure and natural synthesis of cellulose as an individual molecule are critical for forming as a linear chain of glucosyl molecules and self-assemble at the biosynthesis site (McNamara et al., 2015). BC did not solicit the activation of pro-inflammatory cytokines during *in vitro* macrophage screening, while stimulating type II collagen biogenesis (Svensson et al., 2005). BC was indeed found to be a novel *in vivo* degradable scaffold for chondrogenesis (Yadav et al., 2015).

Two distinguished regions of cellulose fibrils are the crystalline and amorphous parts. The chemical processes can be used to produce cellulose nanocrystals (CNCs) by isolating crystalline regions (Tayeb et al., 2018), although mechanical treatments produce cellulose nanofibrils (CNFs) (Moon et al., 2011). Recent use of CNFs and CNCs with nanoscale lateral dimensions has drawn attention, due in part to their natural abundance and biodegradability along with unrivaled flexibility and stiffness, low density, unique rheology, and large aspect ratio (Moon et al., 2011; Lavoine et al., 2012; Hubbe et al., 2017). Cellulose nanoparticles (CNs) refers to all types of cellulose nanomaterials collectively, and are beneficial as substitutes for synthetic petroleum-based adhesives and binders due to their special physiochemical features (Tayeb et al., 2018).

Three-dimensional structuring of bacterial cellulose in an interwoven, translucent, gelatinous, nano-fibrous network of linear polysaccharide polymers occurs at static conditions, as displayed in Gorgieva and Trcek (2019). Compared to cellulose from vegetal sources, bacterial cellulose exhibits extraordinary mechanical characteristics, such as flexibility (Sriplai et al., 2018) and soft-tissue-like stress-strain behavior (Morseburg and Chinga-Carrasco, 2009), along with high levels of crystallinity and water-holding capacity. However, bacterial cellulose is unable to trigger cell attachment or control porosity, and degrades very slowly (Gorgieva and Trcek, 2019). To counteract these drawbacks, chemical and physical modification have been applied both *in situ* and *ex situ* (Gorgieva and Trcek, 2019). Changes to culture media, carbon sourcing, and the inclusion of additional materials occurred *in situ*; physical and chemical treatment of formed BC occurred ex situ (Gorgieva and Trcek, 2019). Bacterial cellulose also features high-purity and net-like morphologies akin to human collagen, benefitting applications in artificial skin, vascular grafts, dental implants, tissue-engineering scaffolds, medical pads, drug delivery, and artificial bone and cartilage creation (Gorgieva and Trcek, 2019).

Cellulose can be used to produce hydrogels with diverse structures and properties due to its abundance of hydroxyl groups (Isobe et al., 2018). Cellulose gel benefits greatly from favorable mechanical properties, thermostability, and biocompatibility, all of which combine to provide it with stiffness (Kobayashi et al., 2014), thermostability (Tsudome et al., 2009), medical applicability (Klemm et al., 2011), and high resistance upon solvent exchange of medium (Isobe et al., 2011). BC is a gel-like substance known as “pellicle,” synthesized by *Gluconacetobacter xylinus*. Despite its excellent mechanical properties (Nakayama et al., 2004) and biocompatibility (Klemm et al., 2011), BC suffers from poor moldability as a result of biological synthesizing activity, restricting it solely to either tube-form or plate-form. In contrast, regenerated cellulose (RC) retains high moldability due to its preparation using a molecular dissolution and coagulation process (Isobe et al., 2018).

## Synthetic Polymers as Scaffolds for Cartilage Tissue Engineering

Synthetic polymers degrade slower than natural polymers due to their higher chemical strength derived from hydrolysable moieties, leading to an extended lifespan in the human body (Table 2) (Hoshikawa et al., 2006). Synthetic materials allow for improved control over mechanical and structural features (Alkaya et al., 2020), and can be easily formed into desired shapes. In fact, synthetic materials such as polyurethane (PU), polylactic acid (PLA), polycaprolactones (PCL), and poly(lactide-co-glycolide) (PLGA) all have high utility as a result of their special properties (e.g., plasticity, degradation rate, and mechanical characteristics) (Ma et al., 2018). Another benefit to synthetic polymers is their immunological inertia, reducing the risk of pathogen transmission (Wang et al., 2011; Yang et al., 2014). These features, combined with their capacity for chemical and mechanical modification and their low degradation rate, pose a significant benefit (Zylinska et al., 2018). However, their use is limited by their harmful acidic degradation products (Lee and Shin, 2007; Camarero-Espinosa et al., 2016; Liu et al., 2016). Other drawbacks include weaker cellular interactions and inadequate intercellular signal transmission compared to natural media (Ge et al., 2012). The polyacid breakdown products also pose a risk of local pH increases at implantation sites (Stoop, 2008). The most commonly used synthetic polymers are currently polyglycolic and PLAs, which are especially commonplace in medical applications (Hoshikawa et al., 2006; Ma et al., 2018).

**TABLE 2:** Characteristics of the outlined synthetic polymers for CTE.

Biomaterials	Symbol	Characteristics	Advantages	Disadvantages	References
Poly(glycolic acid)	PGA	Linear, crystalline hydrophobic polyester; Semicrystalline polymer; Insoluble in most organic solvents	Biocompatibility; Availability; Easy processing; Composited with other biomaterials	Release of acidic degradation products; Poor cell adhesion; Fast biodegradability; Low mechanical properties	Klein et al. (2005), Zwingmann et al. (2007), Nakao et al. (2017), Birru et al. (2018)
Poly(lactic acid)	PLA	Polyesterification reaction production of lactic acid; Lower crystallinity and hydrophilicity than PGA; Four different forms	Biocompatibility, controllable biodegradability; Low toxicity and viscosity; Favorable mechanical properties; Thermostability; Thermoplasticity	Poor cell adhesion	Li et al. (2006), Zwingmann et al. (2007), Lopes et al. (2012), Revati et al. (2017), Smieszek et al. (2019), Szyszka et al. (2019), Marycz et al. (2020)
Poly(ethylene glycol)	PEG	An amphiphilic polymer that cannot be recognized by the immune system	Biocompatibility; Biodegradability; Non-immunogenic; Promoting chondrogenesis; Great flexibility; Low polydispersity	Poor cell adhesion	Karim et al. (2016), Ding and Li (2017), Cheng et al. (2018), Cheng H. et al. (2019), Li et al. (2018), Wang et al. (2019)
Poly-ε-caprolactone	PCL	Semi-crystalline; A synthetic polyester polymer	Biocompatibility; Biodegradability; Elasticity; Excellent mechanical properties; Thermoplastic	Poor hydrophilicity; Poor cell adhesion	Ousema et al. (2012), Sousa et al. (2014), Theodoridis et al. (2019), Venkatesan et al. (2020)

### Polyglycolic Acid (PGA)

Polyglycolic acid (PGA) is a linear, crystalline hydrophobic polyester (Zwingmann et al., 2007). Naturally, hydrolysis of PGA causes its bulk degradation to glycolic acid (Athanasiou et al., 1996). This type of polymer includes poly(hydroxyortho esters) like PLA, PGA, and their copolymers (PLGAs). PGA nonwoven fiber scaffold became widely used to engineer various types of soft tissues, including cartilage (Schaefer et al., 2000), tendon (Deng et al., 2009), blood vessel (Zhang et al., 2007), peripheral nerve and skin (Suzuki et al., 2016). However, the released acidic degradative products of these scaffolds are a major disadvantage that could affect their biocompatibility with seeded cells and host tissues once they are implanted *in vivo* (Lin et al., 2017). MSCs cultured on PGA scaffolds under the effect of LE135, a low molecular weight synthetic inhibitor of the retinoic acid receptor, generated dose-dependent cartilage formation (Ahmed and Hincke, 2014). A composite scaffold composed of PGA-hydroxyapatite (PGA-HA) and autologous MSCs was tested in a rabbit model, resulting in hyaline cartilage and subchondral bone formation (Zhou et al., 2008).

Polyglycolic acid has been successfully used in auricular and laryngotracheal cartilage repair (Klein et al., 2005; Nakao et al., 2017). In a three-PGA-layer construct sandwiched around polypropylene, simulating a 3-D auricular structure, greater cartilage regeneration and angiogenesis were found around the implant (Klein et al., 2005; Nakao et al., 2017). These composite scaffolds were demonstrated to guide MSCs toward cartilage repair, an improvement over microfracturing alone (Erggelet et al., 2009). Klein et al. analyzed the chondrogenic potential of freeze-dried polyglycolic acid-hyaluronan (PGA-HA) implants with preloaded MSCs *in vitro* using a rabbit model and found that MSC-laden PGA-HA scaffolds possess chondrogenic potential and hold promise for stem cell-mediated cartilage regeneration (Klein et al., 2005).

One major shortcoming of PGA materials is the release of acidic degradation products that weaken biocompatibility and trigger inflammatory response. To overcome these shortcomings, PLA/PGA composites have been featured in multiple studies, generating general (Saroia et al., 2018) or anatomic shapes (Lam et al., 2020), and were created by coating fibrous PGA meshes with solutions of PLA in methylene chloride, then evaporating the solvent to deposit PLA on the meshes (Lam et al., 2020). Scaffolds created using this method have been proven to be conducive to cartilage generation both *in vitro* and *in vivo* (Birru et al., 2018).

### Polylactic Acid (PLA)

Polylactic acid is a linear polyester with lower crystallinity and hydrophilicity than PGA (Zwingmann et al., 2007). PLA fibrous scaffolds showed a robust structure and supported the highest proliferation rate of seeded MSCs in physiological solutions (Li et al., 2006). Constructs of MSCs seeded into PLA were investigated in a rabbit model and were shown to form hyaline-like cartilage tissue (Ahmed and Hincke, 2014). Biomaterials composed of PLA polymers are suggested as an engineered scaffold for a variety of medical applications (Marycz et al., 2020), such as bone, cartilage, and peripheral nerve regeneration (Grzesiak et al., 2015; Marycz et al., 2016; Smieszek et al., 2019).

Polylactic acid benefits from its thermostability, slow degradation, good biocompatibility, and low toxicity (Lopes et al., 2012; Li et al., 2015). Furthermore, PLA is known for its low viscosity and good thermoplasticity, making it an outstanding material for 3D printing (Coppola et al., 2018; Dizon et al., 2018). PLA has been approved by the US FDA for clinical use to treat multiple medical conditions, including several orthopedic conditions (Tyler et al., 2016). PLA-based composites with nanohydroxyapatite (nHAp) are highly cell-compatible and are excellent scaffolds for modulating proliferation, viability and differentiation of progenitor cells (Smieszek et al., 2019; Szyszka et al., 2019). The use of *P. purpureum* in PLA matrices as a reinforcement filler offers many benefits. It leads to the production of biocomposites with favorable mechanical characteristics and controllable biodegradability (Revati et al., 2017).

### Poly(Ethylene Glycol) (PEG)

Poly(ethylene glycol) (PEG) is a water soluble polymer that cannot be recognized by the immune system (Cheng et al., 2018), and exists in a variety of structures, including dendritic-like, comb-like, linear, 3-arm, and 4-arm (Karim et al., 2016; Ding and Li, 2017). PEG is typically used to label polymer chains with a molecular weight <20000, while poly(ethylene oxide) (PEO) is the term used for polymers with a higher molecular weight (Li et al., 2018). PEG clears from the body rapidly and has been approved for a myriad of biomedical applications. Furthermore, PEG is capable of transferring these traits to other molecules it is covalently bound to, reversing their toxicity or solubility (Cheng et al., 2018; Li et al., 2018). Cryogelation methods can also be used to create macroporous networks of PEG with interconnected pores in CTE (Cheng H. et al., 2019).

Edward Semple et al. (2016) carried out a large-scale synthesis of linear heterobifunctional PEGs through ring-opening polymerization (ROP) of ethylene oxide (EO) from four initiators composed of azide, alkyne, protected alcohol (*O*-trityl), and protected amine (*N*-dibenzyl). Amphiphilic PEG-based films produced from crosslinking pure amphiphilic PEG copolymers can be modified to alter their mechanical properties by changing the type and quantity of soft and hard segments and adjusting the hydrophilic/hydrophobic ratio (Ji D. et al., 2018; Liu et al., 2018). Soft segments are usually polyesters like PLA and PCL or polyethers such as PEG, which exhibit low glass transition temperatures. Hard segments are typically composed of a chain extender, which links soft and hard segments together by hydrogen bonding (Ji D. et al., 2018).

Poly(ethylene glycols) are hydrolytically non-degradable polymers (Janda and Han, 1996; Gravert and Janda, 1997). and thus minimally metabolized in the body with most polymer chains cleared by the liver (>30 kDa) or kidneys (<30 kDa). PEGs can be readily altered by adding various terminal end groups, such as vinyl ether, allyl ether, or acrylate methacrylate, forming PEG networks. These networks can be made degradable by inserting degradable blocks or crosslinkers (Jain et al., 2017). As such, the degradability and hydrophilicity of polymers can be enhanced via inclusion of PEGs. Because degradation is driven by hydrolysis, hydrophilic PEG content and degradation rate are directly linked (Kutikov et al., 2015).

The fabricated PCL-PEG-PCL (PECE) films seeded with ASCs were able to trigger cell adhesion and proliferation for cartilage formation (Fu et al., 2016). After implanting ASC/PECE films in rats, satisfactory tissue formation was observed (Fu et al., 2016). Saghebasl et al. (2018) developed the thermosensitive and injectable hydrogel PNIPAAm-PECE-PNIPAAm/gel. In comparison to PECE/gel, the inclusion of PNIPAAm raised porosity and increased the swelling ratio, benefitting cell attachment into the scaffold. The PNIPAAm-PECE-PNIPAAm/gel composite is suitable for *in vivo* use at physiological temperatures (37°C) and also found to induce chondrocyte cell growth, expressed cartilage-specific ECM genes, and provided a higher cell survival rate than the PECE/gel composite (Wang et al., 2019).

### Polycaprolactone (PCL)

Synthetic semi-crystalline PCL has recently gained significant attention due to its mechanical strength, elasticity, biodegradability, and biocompatibility (Theodoridis et al., 2019). The FDA approved aliphatic polyester poly(ε-caprolactone) (PCL) presents significant advantages, as the low immunogenic biodegradable compound can mimic the anisotropic and viscoelastic biomechanical features of the articular cartilage (Venkatesan et al., 2020). PCL is also easily processed and chemically versatile, with high structural and thermal stability (Domingos et al., 2012). Its degradation products are also harmless to the human body because they can be metabolized in the tricarboxylic acid cycle (Moura et al., 2020).

While PCL scaffolds can support stem cell differentiation and proliferation, its hydrophobic profile inhibits cellular attachment and thus hurts its suitability in tissue engineering (Sousa et al., 2014). Researchers have attempted to use chemical and plasma treatments together with blending of hydrophilic materials and the ECM to increase the PCL’s hydrophilicity (Sousa et al., 2014; Chen et al., 2016; Silva et al., 2017). PCL-based fibrous scaffolds exhibit high structural integrity following incubation in a physiological solution, and support desirable cell responses of the seeded MSCs (Liu et al., 2006). Chondrogenic differentiation of MSCs on oriented nanofibrous PCL scaffolds was explored in an *in vitro* study. MSCs cultured onto electrospun and oriented PCL scaffolds (500 or 3000 nm fiber diameter) induced chondrogenic markers and enhanced the chondrogenic differentiation of MSCs (Wise et al., 2009).

A 3D woven PCL scaffold seeded with MSCs was fabricated and found to promote chondrogenesis while maintaining favorable mechanical characteristics, without eliciting pro-inflammatory cytokine (Ousema et al., 2012). A biodegradable PCL nanofibrous scaffold seeded with MSCs successfully repaired a swine model of full-thickness cartilage defects (Li et al., 2009). Poly(vinyl alcohol)/polycaprolactone (PVA/PCL) nanofiber scaffolds seeded with BM-MSC were found to support MSC chondrogenic differentiation and proliferation *in vitro* and repaired rabbit full-thickness cartilage defects (Shafiee et al., 2011).

## Decellularized Extracellular Matrix (dECM) May Provide Superior Tissue-Specific Matrix Microenvironment for Tissue Engineering

Cartilage features chondrocytes and extracellular fibers embedded in a matrix, providing strength and acute pliability. The ECM therefore serves as a microenvironment for chondrocytes, and the preservation of stable cellular phenotypes depends on the interaction of cartilage with their pericellular matrix (Gentili and Cancedda, 2009). ECM metabolism also plays an essential role in skeletal tissue development and regeneration/repair, especially in orthopedic diseases and trauma (Gentili and Cancedda, 2009). Its biophysical and biochemical properties are also responsible for the adhesion, nutrition, integrity, migration, and differentiation of individual cells (Ostadal et al., 1995).

### Extracellular Matrix (ECM)

Extracellular matrix is a complex macromolecule network that can undergo self-assembly, and serves as both a reservoir for cytokines and growth factors, as well as a scaffold for tissue and cells (Kresse and Schonherr, 2001). Articular cartilage matrix consists of a strong, dense network of collagen fibers (60% dry weight), primarily type II collagen (80% of the total collagen) interwoven with proteoglycan (PG) (25–35% dry weight) and other non-collagenous proteins (15–20% dry weight) (Azhim et al., 2011). Aggrecan is the largest component of cartilage by percent composition and attracts water molecules, and is responsible for the high shock absorbance of cartilage under load (Gentili and Cancedda, 2009; Maldonado and Nam, 2013).

Extracellular matrix derived from human dermal fibroblasts was found to improve chondrogenesis and stem cell proliferation versus tissue culture plastic (TCP) (Zhou et al., 2016). MSCs-derived ECM (MSC-ECM) was employed as a culture substrate to rejuvenate aged mouse stem cells and enhance their lineage differentiation capacity (Li J. et al., 2014). Porcine synovium-derived stem cell deposited ECM was proven to increase chondrocyte proliferation and delay chondrocyte dedifferentiation (Pei and He, 2012). Cell-derived decellularized extracellular matrix (dECM) has been used as a culture substrate for MSCs to improve cell proliferation and lineage-specific differentiation, and DECM-expanded chondrocytes with enhanced anti-inflammatory properties hold great potential in clinical ACI-based cartilage repair (Yan J. et al., 2020). The chondrocytes seeded onto decellularized human bone marrow derived MSC-ECM (hBMSC-ECM) displayed a significantly greater proliferation rate, maintaining improved chondrocytic phenotype in comparison to the TCP group (Yang Y. et al., 2018). As synovium-derived stem cells (SDSCs) were proposed as tissue-specific stem cells for chondrogenesis, decellularized ECM deposited by SDSCs (SECM) provided an appropriate tissue-specific matrix microenvironment encouraging adult SDSC rejuvenation and improving the regeneration of cartilage (Li et al., 2020).

### Decellularized Extracellular Matrix (dECM)

**FIGURE 2:**
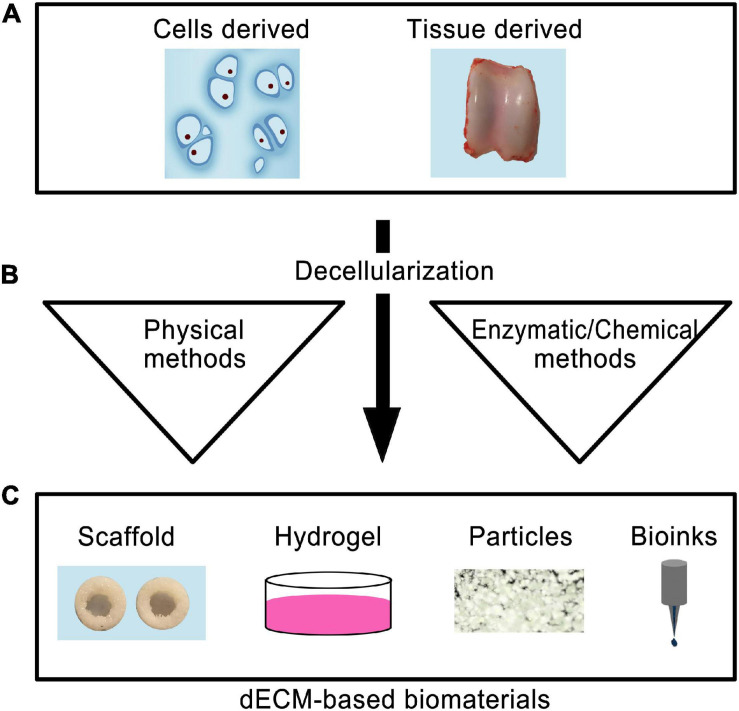
Schematic representation of the use of decellularized biomaterials for cartilage tissue engineering. The cells or tissue-derived biomaterials **(A)** are subjected to decellarization through physical, enzymatic, or chemical methods **(B)**. The resultant dECM biomaterials can be used to produce scaffold, hydrogel, particle forms, or used as bioinks for 3D printing **(C)**.

Using dECM as a scaffold presents several major advantages since the scaffold maintains its original geometry, unlike other processing methods where the dECM is totally pulverized (Kim Y.S. et al., 2019). Depending on the desired topography, composition, and mechanical properties, dECM can be formed from different types of tissue (Yin et al., 2013). However, to achieve these benefits: (a) the dECM must be thoroughly recellularized and; (b) cell debris must be removed from the tissue without destroying collagen fibers, GAGs, and other essential ECM components (Kim Y.S. et al., 2019). dECMs from cartilage tissues (T-dECMs) and cartilage-forming cells (C-dECMs) can be fabricated using enzymatic, chemical, and/or physical methods (Figure 2) (Sun et al., 2018). Both dECM types possess biocompatibility and are able to support chondrogenesis. Notably, cartilage T-dECMs supported chondrogenic differentiation at a higher likelihood than C-dECMs, which more effectively support proliferation and overall chondrogenic differentiation (Sun et al., 2018).

Cartilage T-dECMs with a 3D interconnected porous environment may contribute toward cell proliferation during chondrogenesis and support cellular infiltration. Hyaline and cartilage T-dECMs, both alone (Chang et al., 2014; Rothrauff et al., 2017) or in conjunction with pro-chondrogenic factors (Yang et al., 2008; Schwarz et al., 2012; Rowland et al., 2016), bolstered *in vitro* chondrogenesis of reseeded chondrocytes/stem cells. When implanted *in vivo*, they also led to the repair of cartilage defects and formation of cartilage tissues (Yang et al., 2008, 2010; Kang et al., 2014). Surprisingly, new hyaline cartilage formation also occurred following the *in vivo* implantation of cartilage T-dECMs that had not been supplemented with chondrocytes/stem cells (Grevemeyer et al., 2014; Novak et al., 2016).

### Recellularizing dECM

Scaffolds may increase cartilage regeneration ability if cells are reseeded on decellularized cartilage scaffolds (Xia et al., 2019). Different types of cells have been investigated for cartilage recellularization, including infrapatellar fat pad derived stem cells, bone marrow mesenchymal stem cells (BMSCs), chondrocytes, ASC, and synovium-derived MSCs (Xia et al., 2019). Nonetheless, the decellularization of cartilage and ensuing seeding of cells has proven challenging due to the density of the ECM. Luo et al. (2015) created a channel system in porcine cartilage disks, allowing much improved fluid and cell penetration. Because proteoglycans in cartilage impair cell adhesion (Rich et al., 1981; Yamagata et al., 1989), scientists have also attempted to improve cell adhesion by removing GAGs from cartilage tissue (Elsaesser et al., 2014). For instance, Bautista et al. (2016) incorporated chondroitinase ABC during decellularization with the intent of removing GAGs from porcine articular cartilage. While successful in enhancing decellularization, the treatment did not improve recellularization rates. Furthermore, dECM scaffolds cannot be readily scaled despite their strengths and ability to sustain a native architecture although highly porous dECM scaffolds with different geometries have been produced with methods such as freeze-drying in order to overcome this barrier (Chen et al., 2015; Gawlitta et al., 2015).

### Hydrogels (HG)

Hydrogels (HG) are a class of soft materials comprised of crosslinked polymer chains arranged in a porous 3D network, which are known for their ability to hold up to 99.9% water by weight. They are fabricated from either synthetic or natural sources and possess good mechanical properties together with unique biocompatibility (Curvello et al., 2019). Several hydrogels have been utilized extensively in recent decades, with varying composition, structures, and properties. HGs have been introduced in medical applications such as biosensors, contact lenses, drug delivery devices, and artificial implant linings (Meng et al., 2019), as well as wound dressings, scaffolds, and hygienic products (Alves et al., 2016; Jung et al., 2017).

Hydrogel scaffolds exhibit compressive strength and allow load transfer from the environment to chondrocytes (Spiller et al., 2011). Hydrogels mimic the physical and chemical conditions of the extracellular matrix, promoting cell differentiation and multiplication along with bolstering integration with trauma sites (Arakaki et al., 2010). Combinatorial hydrogels are a class of hydrogels that allow for the study of cellular responses to multiple biophysical (e.g., crosslink density) and biochemical (e.g., ligand tethering) signals (Benmassaoud et al., 2020).

Natural hydrogel constructs are often composed of polysaccharide or protein chains. Polysaccharides have beneficial hydrophilic structures that enable the creation of hydrogels from many different biomaterials, such as dextran, chitin, alginate, chitosan, cellulose, starch, pectin, xanthan gum, and HA. Synthetic polymers such as polyacrylamide, PVA, PEG, and PEO have all been used for hydrogel formation. While synthetic photopolymerizable hydrogels are cell-compatible, the presence of long-lasting polymer components compromises their mechanical performance and hinders cell migration (Lee et al., 2006; Roberts and Bryant, 2013).

Hydrogels can promote chondrogenic potential and allow for *in situ* scaffold formation without open surgery. Solutions can be injected intra-articularly without affecting chondrocyte colonization or cartilage differentiation (Li S. et al., 2019). Solid supporters can be added to boost the mechanical stability of hydrogels (Huang et al., 2014). Hydrogels produced with artificial polymers, such as PCL, PLGA, PEG, and polymethyl methacrylate (PMMA), can reach mechanical strengths of 20–120 MPa (Pahlevanzadeh et al., 2018). Polymeric blending of natural and synthetic polymers can produce hydrogels with differing chemical and physical properties for various biomedical applications (Li L. et al., 2019).

## Fabrications and Preparation Techniques of Biological Scaffolds

Scaffolds are clinically superior to scaffold-free environments, forgoing invasive surgical procedures for tissue extraction from patients. Additionally, scaffolds provide increased control over the filling of cartilage defects and can reduce patient recovery times (Caron et al., 2012). Cell migration and adhesion are influenced by the microarchitecture and geometry of scaffolds (Oh et al., 2010) as well as pore size. Pores should be small enough to give cells a sufficient surface area for adhesion, yet large enough to enable ECM production and cell migration (Chen et al., 2006). Porous 3D scaffolds are a prominent option for tissue engineering because they mimic *in vivo* physiological microenvironments closely. Their excellent porosity also enables cell growth, migration, and adhesion by providing necessary nutrition and transporting metabolic waste (Ma et al., 2018). As a result, the selection of a 3D scaffold is important when determining how the scaffold will behave in different tissue engineering applications (Ko et al., 2010). The differences between scaffold preparation processes have significant influence on the shape, size and porosity of the hole in the support, which may directly affect the migration, differentiation and proliferation of seed cells (Fu et al., 2018). Advances in manufacturing have resulted in 3D printing as a leading technology for producing tunable scaffolds in the field of TE (Cheng A. et al., 2019).

### Three-Dimensional (3D) Bioprinting Techniques

**FIGURE 3:**
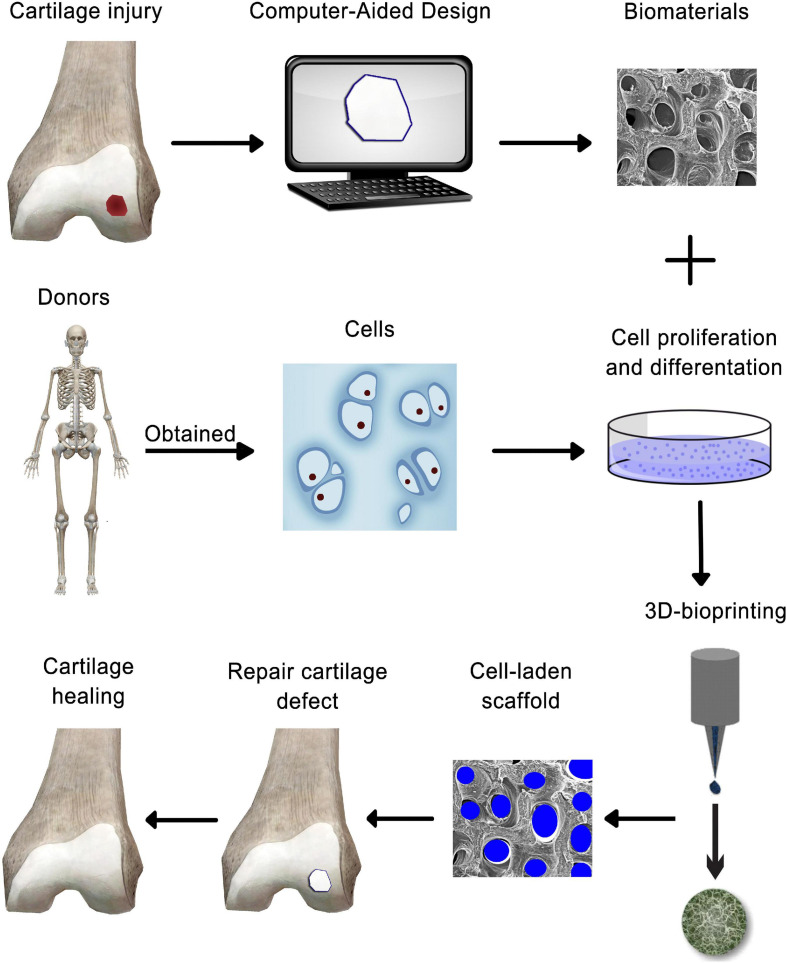
Schematic representation of 3-D bioprinting technology-based cartilage tissue engineering.

Cartilage is an avascular, alymphatic, and aneural tissue with limited self-renewal, which makes cartilage a relatively simple tissue for regenerating damaged cartilage through bioprinting approaches. Moreover, its organized zonal cell matrix distribution and density makes it suitable for duplication by 3D bioprinting (Figure 3) (Ji X.F. et al., 2018). Bioprinting offers great prospects of printing tissue analogs through the delivery of live cells with suitable material in a defined and organized manner (Derby, 2012). The concept of bioprinting is essentially an extension of the idea that uses additive manufacturing (AM) methods for building up 3D tissue structures layer-by-layer (Kundu et al., 2015), from the bottom up, with high-resolution deposition of materials and cells as well as a customized inner structure, with the aim of duplicating the complexity of native tissues or restoring damaged structure and functionality (Ji X.F. et al., 2018). This advancement has made custom patient-tailored product fabrication possible as MRI and CT images can now be used to create personalized solutions (Roseti et al., 2017).

Three-dimensional bioprinting is a modern method for 3D living tissue/organ structure fabrication using “bioinks” (Buckwalter et al., 2004; Levingstone et al., 2016). A wide range of bioinks have been developed for micro-extrusion and inkjet bioprinting, including agarose, GelMA, alginate, silk, collagen, fibrin, and forms of poly(ethylene) glycol (PEG) and HA (Daly et al., 2017). The two primary bioink categories are scaffold-based and scaffold-free. Scaffold-based bioinks include cells and biomaterials, creating a scaffold with structural support for cell differentiation and growth (Roseti et al., 2017). Scaffold-free bioinks are composed of aggregates such as tissue strands, cell pellets, and spheroids that secrete ECM (Gruene et al., 2011). Scaffold-based bioinks are more common, although both types can complement the other’s strengths and weaknesses (Ozbolat, 2015).

Conventional “subtractive methods” remove materials from an initial block using a top-to-bottom approach, in which cells are seeded onto the finished scaffold at a later time (Bishop et al., 2017; Ngo et al., 2018). Due to the intrinsic nature of bioprinting, finer control over cell spatial distribution can be achieved, producing more homogenous scaffolds that better support cell viability (Roseti et al., 2018).

3D bioprinting typically begins with a computer-assisted design/model for depositing live cells and biomaterials onto a new 3D biostructure, after which post-processing results in the maturation of these cell-laden constructs (Murphy and Atala, 2014). A chondro-inductive bioink was created by combining alginate, gellan, and cartilage ECM particles (Kesti et al., 2015). The ink was capable of printing highly accurate anatomical shapes, and matrix components were produced successfully (Daly et al., 2017). In a novel scaffold-free bioprinting method, modular cartilage tissue strands generated by fusing tissue spheroids in a confining mold were capable of being printed into 3D constructs using a robotic dispensing system (Yu et al., 2016).

The most prevalent scaffold-based 3D bioprinting technologies today are based on laser technology, extrusion, and jetting (Table 3) (Roseti et al., 2018). Jetting-based 3D bioprinting can be conducted using either a continuous inkjet or using specifically distributed deposition of single droplets (drop-on-demand) (Irvine and Venkatraman, 2016). The drop-on-demand method includes three distinct droplet generation strategies: electrostatic, piezoelectric, and thermal (80–90%) (Roseti et al., 2018). A novel bioprinting approach was developed to print ovine MSC constructs submerged in oil (Roseti et al., 2018).

**TABLE 3:** Comparison of the three types of 3-D bioprinting techniques.

	Jetting-based	Extrusion-based	Laser-based
Printer cost	Low	Moderate	High
Biomaterial viscosity	Medium	High	Medium to high
Print speed	Fast (1–10,000 droplets/s)	Slow (10–50 μm/s)	Medium-fast (200–1,600 mm/s)
Cell viability (%)	80%–90%	40%–95%	95%
Resolution	High (up to 50 μm)	Moderate (100 μm to millimeters)	High (10–50 μm)
Cell densities	Low (≤106 cells/ml)	High (cell spheroids)	Medium (≤108 cells/ml)
Quality of vertical structure	Poor	Good	Fair
Advantage	High cell viability; High printing speeds; Low cost; Wide availability; Easy operation	High cell densities; High cell viability; Broad selection of biomaterials; High deposition rates; High print speeds; Anatomically correct porous construct generation	Nozzle free; Fast and accurate fabrication; High resolution; High precision; High cell viabilities
Disadvantage	Low droplet directionality; Nozzle clogging; Limited biomaterials selection; Low cell density and concentration of the ink; Heat and sheer stresses induced damage to cells	Low resolution; Deformation; Encapsulated cell apoptosis; Low cell viability	High cost; Low speed; Low built capability; Possible cytotoxicity; UV induced DNA damage; Low stability and scalability; Limited printing directionality
Tissue engineering application	Blood vessel, bone, cartilage, neuron, liver	Blood vessel, bone, lungs, liver, cartilage, neuron, muscle, ear, skin, lipid bilayers	Blood vessel, bone, skin, adipose, cardiac tissue
References	Irvine and Venkatraman (2016), Roseti et al. (2018), Roseti et al. (2017)	Cole et al. (2009), Hasan et al. (2014), Daly et al. (2016), Pillai et al. (2018), Dhawan et al. (2019)	Roseti et al. (2018), Dhawan et al. (2019), Irvine and Venkatraman (2016)

Laser-based 3D bioprinting is a complex, expensive technique involving the use of pulsed laser energy to transfer materials to a receiving substrate (Roseti et al., 2018), which can initiate a two-layer droplet release (Dhawan et al., 2019). The top layer comprises an energy absorbing donor layer, whereas the bottom layer is the selected bioink. Bioink droplet release occurs after emission of the laser pulse onto the top donor surface layer, producing a bubble at the interface between the layers and propelling the droplet onto the substrate (Dhawan et al., 2019). This method avoids mechanical stress from direct contact with the printer and has greater resolution and precision than other options. The biggest obstacle is the cost of laser-based systems (Dhawan et al., 2019). The size and complexity of the required equipment also limits its usage, along with its inferior cell viability relative to inkjet mechanisms (Irvine and Venkatraman, 2016).

Extrusion-based bioprinting is a pressure-based bioprinting method currently employed in the fabrication of heterogenous scaffolds for osteochondral regeneration (Dhawan et al., 2019). This technology is the basis for most commercial bioprinters, and uses a micro-nozzle to dispense bioink filaments via pneumatic or piston pressure. This system is run by mechanical, solenoid, or pneumatic control. A wide variety of bioinks, such as tissue spheroids, hydrogels, tissue stands, and microcarriers, can be used due to the large, flexible nozzle size. This technique has been applied successfully to print several types of tissues, such as lungs, cartilage, liver tissue, and lipid bilayers (Hasan et al., 2014; Pillai et al., 2018). This technique can release highly viscous bioinks with the micro-nozzle, raising deposition rates and printing speed and enabling the use of synthetic polymers (Dhawan et al., 2019). Other strengths include production scalability, high cell viability, and anatomically correct porous construct generation (Dhawan et al., 2019). However, high pressure associated with mechanical extrusion poses drawbacks such as low resolution, deformation, and encapsulated cell apoptosis (Cole et al., 2009; Daly et al., 2016). Nonetheless, the mechanical qualities of bioprinted constructs are subpar compared to those of the native tissue and require further optimizations.

## Conclusion and Future Directions

Effective repairing of damaged cartilage tissues caused by high impact sports or diseases remain a major clinical challenge. CTE provides a promising avenue to this unmet challenge. Significant progresses have been made in CTE, including the identification and the use of chondrogenic biofactors, the isolation and characterization of chondrogenic progenitors from various sources, and the development of cell-friendly, biocompatible scaffold materials. The rapid advance of 3D-bioprinting techniques further facilitates the clinical realization of CTE. Nonetheless, many hurdles remain ahead of successful and effective CTE. At the basic research front, we need a better understanding of cartilage biology, including the layered/zoned structures with different organization, and the distinct morphologies and functionalities of chondrocytes in different layers/zones. Efforts have to be devoted to the characterization and optimization of efficacious chondrogenic biofactors. Another challenge is to establish reliable techniques to generate sufficient chondrogenic progenitor cells from various cell sources, including MSCs from different tissues or effectively directed chondrogenic differentiation from iPSCs. As discussed above, many biocompatible scaffold biomaterials have been developed; but vast majority need to be rigorously tested in pre-clinical studies and clinical trials. Ultimately, a 3D-bioprinting fabrication may offer the opportunity to assemble chondrogenic factor-stimulated and progenitor cell-loaded scaffold biomaterials to accomplish effective regeneration and repair of injured cartilage.

## Author Contributions

All authors contributed to the article and approved the submitted version.

## Conflict of Interest

The authors declare that the research was conducted in the absence of any commercial or financial relationships that could be construed as a potential conflict of interest.
